# Biocomposites with Epoxy Resin Matrix Modified with Ingredients of Natural Origin

**DOI:** 10.3390/ma15207167

**Published:** 2022-10-14

**Authors:** Piotr Szatkowski, Martyna Szatkowska, Jacek Gralewski, Leszek Czechowski, Slawomir Kedziora

**Affiliations:** 1 Faculty of Materials Science and Ceramics, AGH University of Science and Technology, 30-059 Krakow, Poland; pszatko@agh.edu.pl (P.S.); szatkowska@agh.edu.pl (M.S.); 2 Institute of Marketing and Sustainable Development, Lodz University of Technology, 93-590 Lodz, Poland; jacek.gralewski@p.lodz.pl; 3 Department of Strength of Materials, Lodz University of Technology, 90-537 Lodz, Poland; 4 Faculty of Science, Technology and Medicine, Luxembourg University, L-1359 Luxembourg, Luxembourg; slawomir.kedziora@uni.lu

**Keywords:** cellulose, biocomposites, natural fibers, epoxy resin

## Abstract

This study aims to present various forms of cellulose, whose shape depends on the source of origin, and to demonstrate the differences in the influence on the properties of materials produced with its participation. For this purpose, composites with various plant additives have been designed and obtained. Some of them have undergone chemical and pyrolytic modifications. The results of the mechanical, physicochemical and microscopic tests showed differences in cellulose structure, even in the case of very similar sources, and its diversified influence on the characteristics of the obtained materials. The research shows the effect of the use of natural additives and their modified versions on the mechanical properties of the composite based on epoxy resin. It turns out that cellulose modifiers are not only fillers that reduce the price of the final product but can also increase some mechanical properties, e.g., compressive strength, which is an additional advantage and a reason for wider use. The potential of natural resources is not yet fully understood. Relatively recently, people have started to be interested in cellulose on a nanometric scale, as it turns out that it can exist in several different forms with interesting properties.

## 1. Introduction

**Figure 1:**
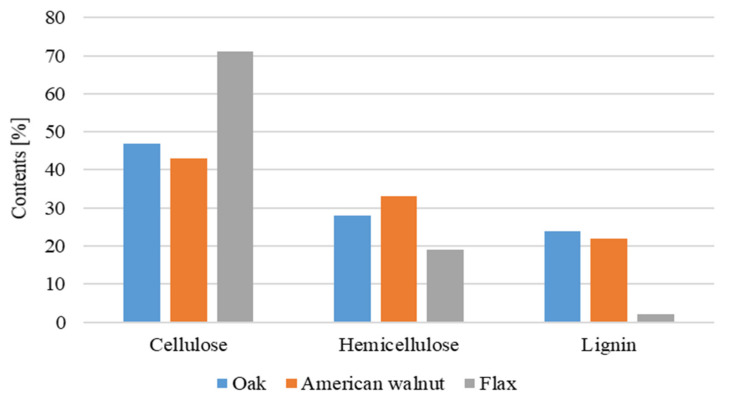
The content of cellulose, hemicellulose and lignin in oak, American walnut and flax [2,26,27,28].

In the industry, composites obtained with the participation of renewable raw materials can be found more and more often [1,2,3,4,5,6,7]. The interest in using modifiers of natural origin is increasing due to people’s growing awareness of environmental protection and the EU’s legal limitations [8]. The reinforcement can be made of natural fibers from various parts of the plant [8,9], leaves [10,11,12], stems [13,14,15], fruits [16,17,18] or seeds [19,20]. In order to expand the biocomposites market, solutions are sought to improve durability, dimensional stability, water resistance and strength properties [21,22]. The fibers differ in their mechanical properties, which are influenced by inter alia, the chemical composition, cellulose content and the orientation of the fiber fibrils [2]. Cellulose fibers have different forms (shapes), i.e., long and short fibers, cellulose dust and cellulose nanoforms [23,24,25]. Plant fibers are chemically composed of cellulose, hemicellulose and lignin as well as pectins, proteins, soluble compounds and inorganic substances, which together constitute the building composition. A single fiber is made of macrofibrils, in which cellulose microfibrils act as reinforcement and hemicellulose with lignin as the matrix. The properties of the fibers depend on the cellulose content and the angle at which the microfibrils are arranged in relation to the fiber axis. The tests carried out on lignin-free fibers bring to the conclusion that the mechanical properties (Young’s modulus) decrease with the increase in the helical angle, as shown in Figure 1 [2,26,27,28].

The list does not include horsetail (due to the lack of literature data), for which the cellulose content is the lowest and amounts to about a dozen percent. A secondary motive for choosing horsetail as one of the modifiers was that it is a raw material with a relatively high silica content compared to other plants. The content ranges from 3% to 5% (dry weight). The silica present in the walls of the plant, as in cellulose, is responsible for its strength [6]. Cellulose is one of the most promising environmentally friendly modifiers and is present in the highest amounts. It can be used as a component of a composite [29,30] or as a cellulose-cellulose composite [31].

The use of plant raw materials in composites generates difficulties with compatibility. At the interface, we have a combination of a hydrophobic matrix with a hydrophilic filler [32,33]. In order to improve adhesion, physical modification can be used, resulting in changes on the surface of the fibers, which leads to the improvement of a mechanical bond. These methods include stretching, calendering, thermal and plasma treatment [34]. Another type of modification is chemical methods [35,36], aimed at changing the hydrophilic nature of the fiber. They are carried out with the participation of surface functional groups. These include micerization (with NaOH solution) [10], silanization (with silicon compounds-aminosilanes), acetylation (with acetic anhydride), benzoylation (with benzoyl chloride), acrylation (with acrylic acid) and modification with carboxylic acids [2,37,38].

The research aimed to produce and perform the biocomposites tests with a polymer matrix and a plant-based modifying phase and also to check how the content of cellulose and its modification affect the properties of the composite. The literature currently provides information on epoxy composites reinforced with natural short fibers and microfibers from cereal hulls. Urbaniak, in [8,39], tested the 40% content of the modifying phase. Faruk et al., in [2], presented natural fibers and their possible modification. In this article, we can read about the modification of epoxy resin with natural fibers and their modifications in the proportion by weight of 2.5 and 10%. The given values have been chosen because no such additions have been encountered in the literature. A choice of 2.5% gives a picture of the effect of a small amount of additive. This is a value that should not have a significant effect on the resulting composite properties. The obtained values of the examined properties should show the trend in which the changes of these properties go, and 10% allows us to determine the properties of the average amount of additives. It will make it possible to determine the effect of the additive on the properties of the obtained compositions and provide confirmation of changes and trends in properties obtained with the content of 2.5%. Using a larger amount of biofiller would significantly impact the production technology and the obtained properties (e.g., 20–40%).

## 2. Materials and Methods

### 2.1. Materials

Epidian 652 epoxy resin cross-linked with IDA hardener was used as the matrix (Ciech, Nowa Sarzyna, Poland). This resin is characterized by low viscosity and transparency and shows minimal shrinkage during cross-linking. The modifying phase was chosen to have different forms of cellulose:

- Microcrystalline cellulose from manufacturer Sigma-Aldrich (Saint Louis, MO, USA), in the form of powder.
- Short linen fibers made of Safilin roving (tex 2000).
- Oak wood flour and American walnut wood flour, waste from the carpentry workshop, not contaminated with other types of wood.
- Chemically treated oak wood flour.
- Field horsetail, dried and powdered leaves and stems, harvested from rural areas (own cultivation, Krakow, Poland).
- Powdered field horsetail subjected to pyrolytic treatment (own cultivation, Krakow, Poland).

### 2.2. Production

**Figure 2:**
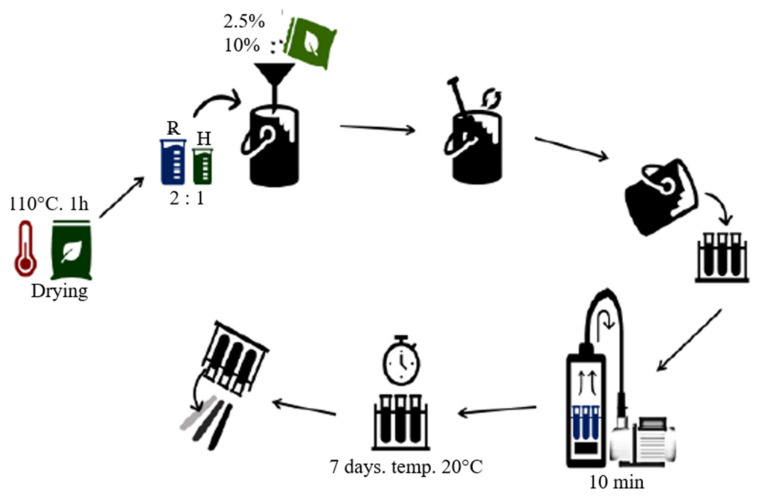
Stages of composite production.

The test specimens were produced by the casting method with the use of a vacuum pump (−0.8 bar). The process is shown schematically in Figure 2 and is described in detail below. The materials produced are summarized in Table 1. Plastic test tubes were used as the molds, which made it possible to obtain cylindrical samples. 

**Figure 3:**
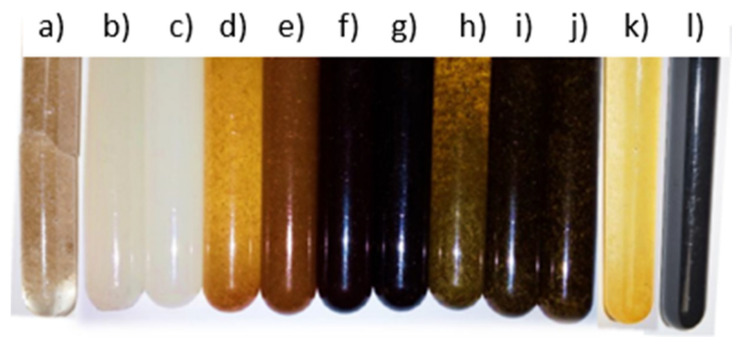
The pictures of obtained biocomposites. From the left with: flax (**a**), cellulose (**b**,**c**), oak wood flour (**d**,**f**), American walnut wood flour (**e**,**g**), horsetail (**h**–**j**), modified oak wood flour (**k**), modified horsetail (**l**).

The first stage of the sample production process was the preparation of natural components. The flax roving was cut into short fibers. The dried horsetail was ground to a powder form, and then the ground horsetail and wood flour were placed in the dryer at 110 °C for 1 h in order to completely remove the moisture. Subsequently, the appropriate amount of the additive was added to the prepared portion of the resin, mixed thoroughly, and then a portion of the hardener was added and thoroughly mixed again. The resin and the hardener were combined in a 2:1 ratio (stoichiometric ratio established by the resin manufacturer). The third stage was based on the mixture poured into molds, which were then placed in a glass tube and a vacuum chamber. The pipe, with a valve enabling the creation of the vacuum, was closed with a rubber stopper and ended with a rubber hose connected to an oil vacuum pump. After switching on the pump, air was sucked from inside the pipe, including samples, which were observed as foam accumulating above the surface of the uncured resin (the air released from composites). The activity was repeated several times for about 10 min. The samples obtained in this way were allowed to cross-link at room temperature for 7 days, the time necessary to obtain the maximum mechanical properties of the matrix. Figure 3 shows all the samples (12) produced.

**Figure 4:**
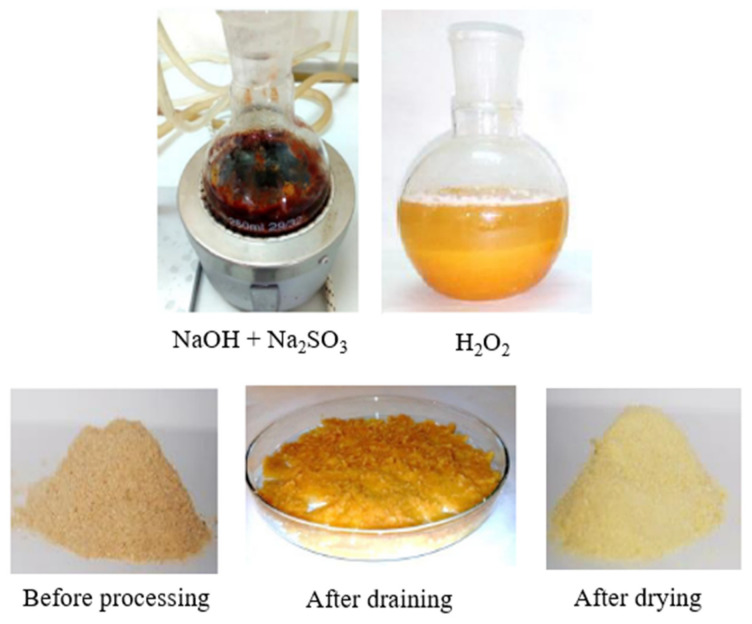
Stages of chemical modification.

**Figure 5:**
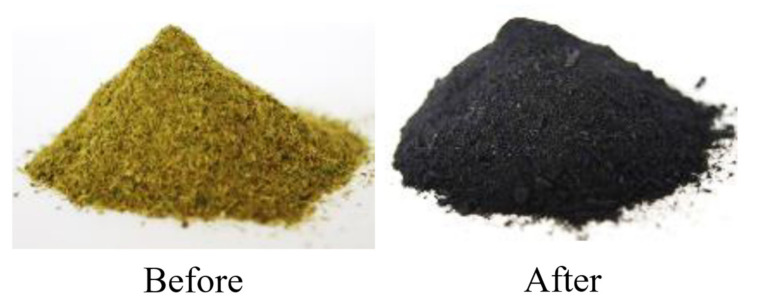
Material before and after modification.

Chemical modification of the material was performed in order to remove lignin, pectins and waxes from the material, according to the recipe presented by the team of Mingwei Zhu [11]. NaOH (2.5 mol/dm^3^) and Na_2_SO_3_ (0.4 mol/dm^3^) were dissolved in distilled water, and wood flour was added. The solutions were heated to a boil and then boiled for 3 h. After this time, the supernatant solution was removed, and the residue was taken up in 30% hydrogen peroxide. After 30 min, the operations were repeated, the solution was decanted from the meal and hydrogen peroxide was added. After decanting the solution, the resulting material started to dry. The subsequent stages of modification are shown in the form of photos in Figure 4. The powder was carbonized at 1000 °C, presented in Figure 5 in the form of photos before and after the modification.

In order to determine the mechanical properties, the behavior of the samples during the three-point bending test and the compression test was assessed. The bending test was carried out on a Zwick 1435 machine (Zwick: Geisa, Germany) according to PN-EN ISO 178. The support spacing was set at 35 mm. The compression test was carried out on a TIRAtest 2300 testing machine (TIRA GmbH: Schalkau, Germany) according to PN-EN ISO 604:2006. The sample was compressed at a speed of 1.00 mm/min. The evaluation of mechanical properties (fracture toughness) under dynamic loads was made based on an impact test carried out with a Charpy pendulum hammer (VEB Werkstoffprüfmaschinen Leipzig, Leipzig, Germany). Unnotched samples with an area of 1.33 mm^2^, placed at a distance of 0.38 m from the axis of the hammer pendulum, were broken with one hammer blow, weighing 6.915 kg. Microscopic observations of plant additives and produced biocomposites were carried out using the NOVA NANO SEM 200 (Thermo Fisher: Waltham, MA, USA) scanning electron microscope (cooperating with the EDS analyzer) and the EDS microanalysis, allowing the identification of chemical elements included in the tested samples. Since the samples analyzed with the scanning microscope must be electrically conductive, it was necessary to spray the samples with carbon before proceeding with the test. The specimens were attached with a carbon tape, which ensures the discharge of charges from them. Plant components were subjected to microscopic observations using the KEYENCE VHS digital microscope (Keyence: Osaka, Japan). The thermal stability was determined based on the thermogravimetric analysis (TG) and the differential thermogravimetric analysis (DTG). Measurements were made using a NETZSCH STA 449 F3 thermal analyzer (AZoM: Bellevue, WA, USA) in a nitrogen atmosphere. The samples, weighing about 10 mg, were placed in an Al_2_O_3_ crucible and heated from room temperature to 1000 °C at a rate of 10 °C/min. The density of the obtained materials was determined by geometric and hydrostatic methods (taking into account the porosity). Infrared spectroscopy (FTIR) was carried out using a BRUKER TENSOR 27 spectrometer (Keyence: Osaka, Japan) with the attenuated total reflection (ATR) technique with a diamond crystal in the mid-infrared range of 4000–600 cm^−1^, with a resolution of 4 cm^−1^ and the number of scans of 64.

## 3. Results and Discussion

### 3.1. Characteristics of Natural Additives Based on the Microstructure and EDS Research

**Figure 6:**
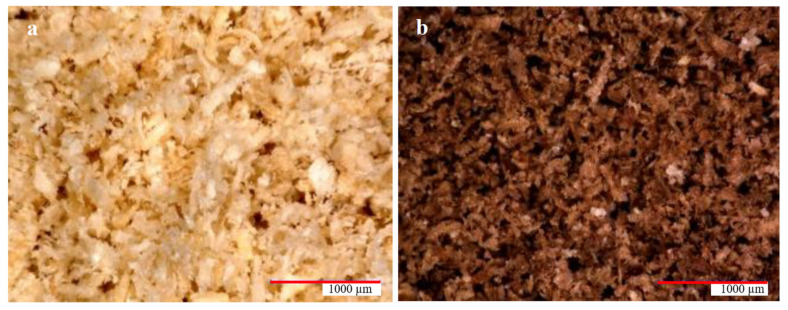
Microscopic image (magnification 50×) of (**a**) oak wood flour and (**b**) American walnut wood flour.

Figure 6 shows wood flour using a digital microscope. Apart from the differences in color (which depend on the type of wood, the dyes they contain, as well as various external factors, such as the climate in which the tree grew or the conditions to which it was exposed after cutting), magnification does not allow significant differences to be seen between both extracts. The powder particles appear to be of similar shape and size. There have been many studies on the shape of molecules and their properties [40].

**Figure 7:**
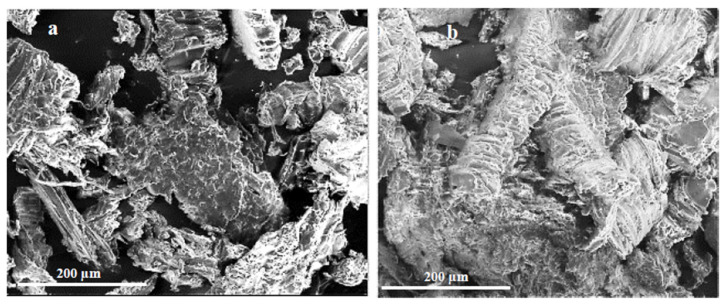
SEM microscopic image (magnification 350×) of (**a**) oak wood flour and (**b**) American walnut wood flour.

In order to compare the wood flour, SEM photos were additionally taken (Figure 7). They revealed differences in the microstructure of the meals, although it would seem that they would be similar due to the affinity of their source. In the SEM images of oak flour, individual elements of the powder can be seen, which take the shape of thin flakes with slightly jagged edges and smooth surfaces, while those of walnut wood resemble flakes/bars with a wavy surface and sharp, jagged edges.

**Figure 8:**
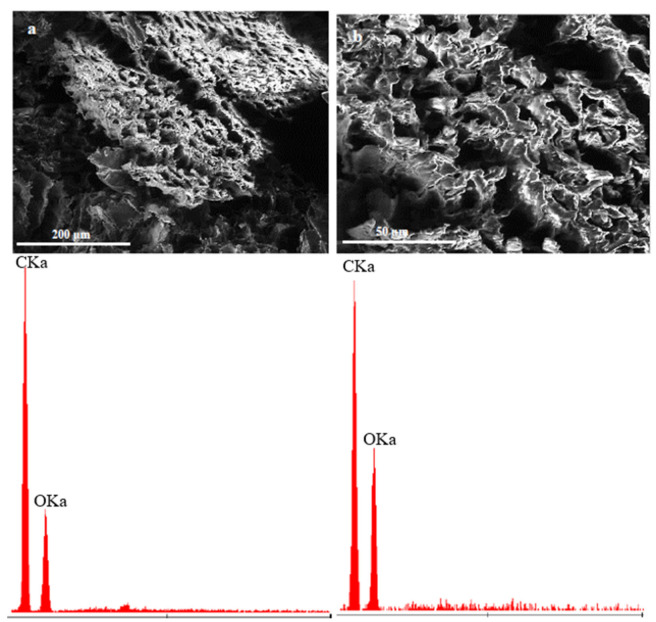
Wood flour before chemical treatment (**a**)—magnification 350× and after chemical treatment (**b**)—magnification 1000× and below adequate EDS results.

**Figure 9:**
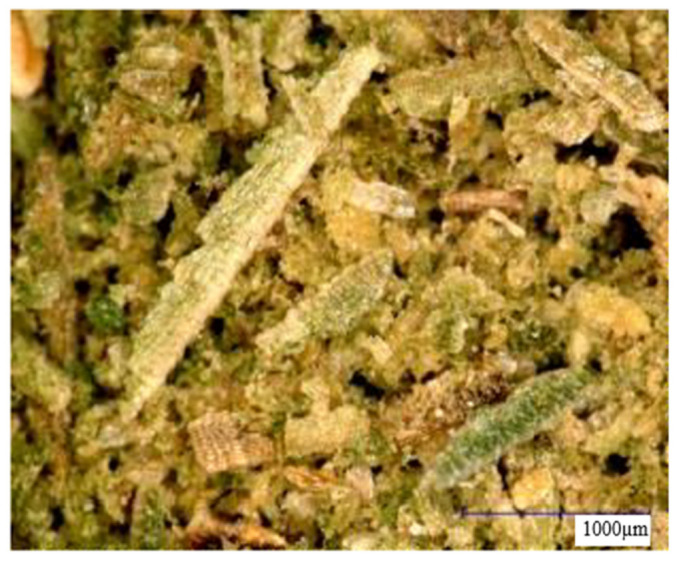
Horsetail (magnification 50×).

**Figure 10:**
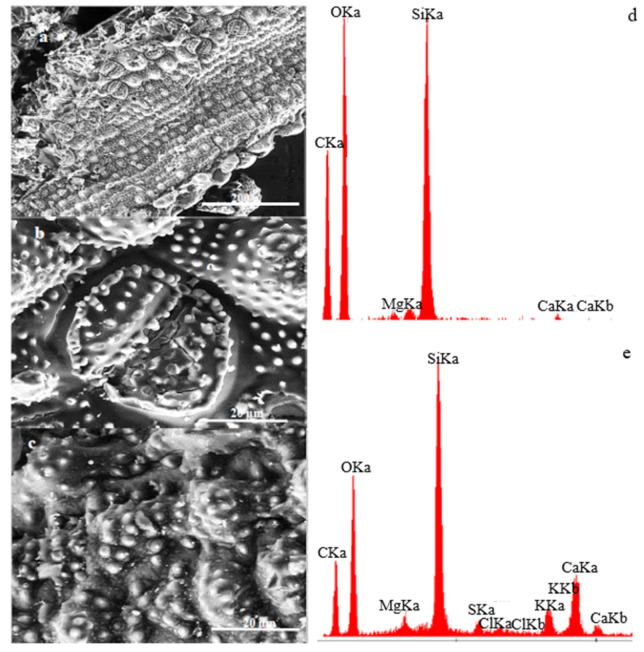
Horsetail–SEM (**a**)—350×, (**b**)—3000×, (**c**) horsetail after carbonization—3000×, (**d**) horsetail EDS before carbonization, (**e**) horsetail EDS after carbonization.

The microscopic photos shown in Figure 8 present a comparison of unmodified (a) and modified (b) wood flour. The modification was aimed at removing lignin. (Lignin removal was required to check the impact of lignin on the properties of additives. When the lignin was removed, then our wood flour contained cellulose fibres without matrix.) The photo shows porous structures with cellulose fibers. The EDS result confirms that the applied modifier has carbon and oxygen (and hydrogen) in its composition. It leads to the conclusion that the substrate was not contaminated. Another material used as the modifying phase is powdered horsetail. In comparison with wood flour, it is characterized by larger particles and shapes with sharp edges. Its microstructure, shown in Figure 9, presents a fragment of the horsetail stalk/leaves. Its entire surface is covered with numerous hemispherical protrusions with smaller hemispherical/conical protrusions. The analysis presented in Figure 10 is horsetail before and after modification. Despite the application of the temperature of 1000 °C, the microstructure was not damaged, and on the surface of the modified horsetail, one can observe protrusions with silica crystals.

**Figure 11:**
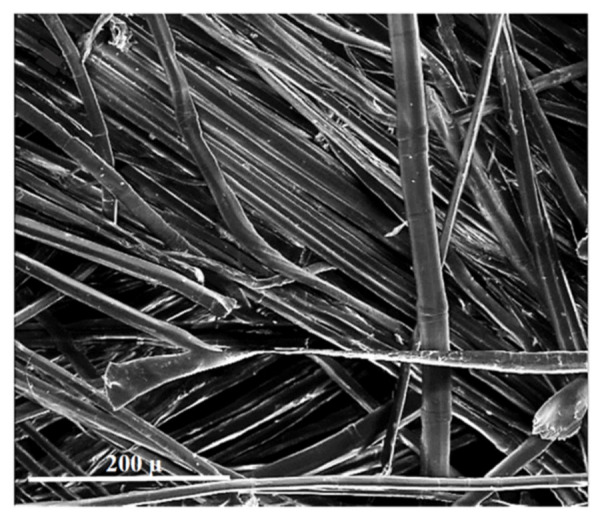
Flax fibers (own research).

Figure 11 shows SEM images of flax fibers, where one can observe randomly arranged individual fibers, some of which are fibrous or twisted. Among the modifiers used, their transverse dimension is the smallest, and the longitudinal dimension is the longest.

**Figure 12:**
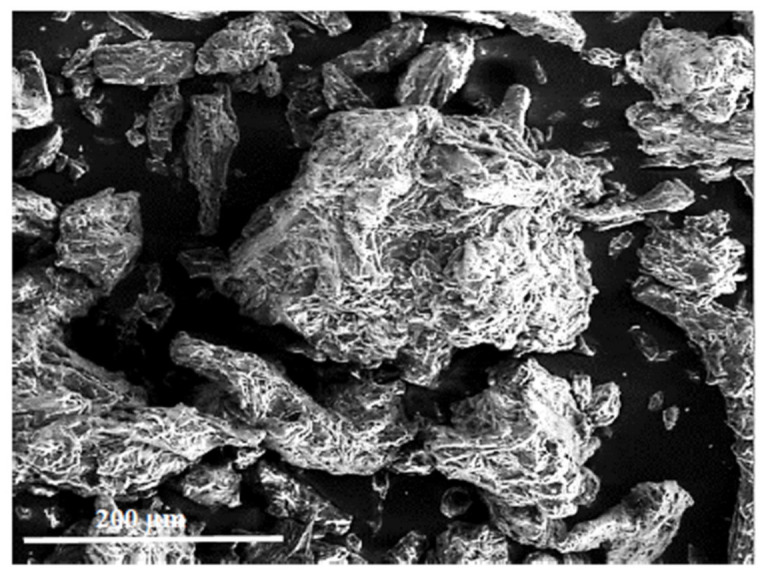
Microcrystalline cellulose (magnification 350×) flax.

The last of the additives used was microcrystalline cellulose, the SEM image shown in Figure 12. This form is characterized by different crystal sizes, being an agglomerate of smaller elements. The surface of the crystals is uneven with sharp edges.

### 3.2. Flexural Strength and Modulus of Elasticity in Bending

**Figure 13:**
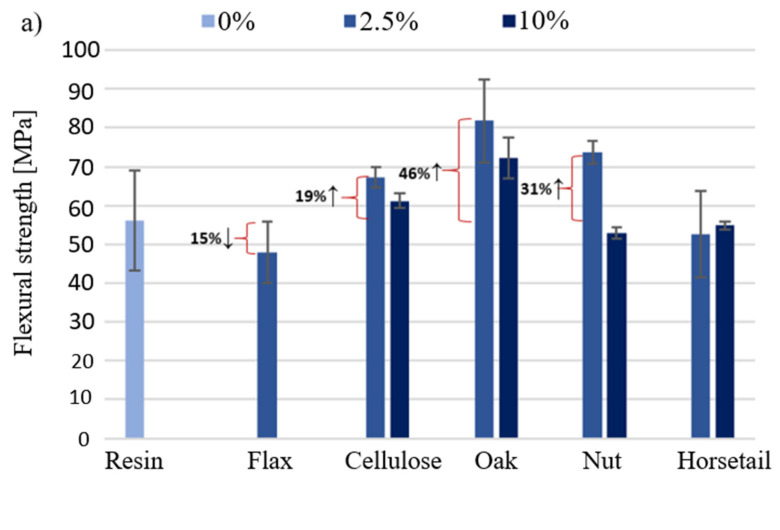

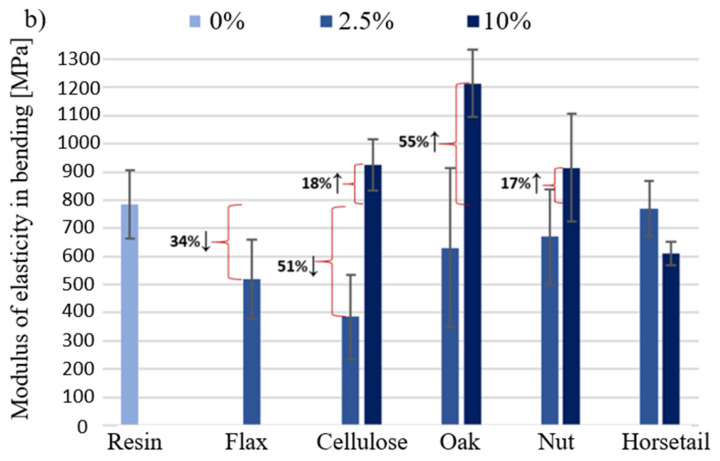
Bending strength (**a**) and bending modulus (**b**) of the obtained.

Figure 13 shows the results of the bending strength and modulus of elasticity determined during the bending test of the produced biocomposites.

Figure 13a shows the bending strength of biocomposites with a given component. The greatest increase in bending strength was recorded for the addition of oak wood flour (46% ↑−2.5%). This relationship applies to both modifier percentages. There was a decrease in the tested value for flax fibers (15%) and a slight decrease for horsetail. It is worth noting that only in the case of horsetail, 10% of the additive shows a higher bending strength than the 2.5% share of the additive. According to the manufacturer’s data, the Epidian 652 resin has a flexural modulus of 2380 MPa. The obtained results for the pure resin and the composition with natural additives are much lower and amount to an average of 25% of this value for the addition of 2.5% of the modifier and 38% for the 10% additive. The introduction of modifiers in the amount of 2.5% to the matrix causes the appearance of places in the sample where the stresses caused during bending are partially dissipated in the material; this effect is lacking in the resin. The use of additives in the amount of 10% reduces the strength in relation to 2.5%, because this amount is already too high and the samples may form poorly filtered matrix agglomerates, which will be the weakest parts of the sample. Interpreting the results presented in Figure 13b for samples with less fillers, it could be concluded that they reduce the material’s stiffness. However, the obtained results are burdened with relatively large deviations of values, which may be caused by sample defects triggered by insufficient cross-linking of the epoxy matrix. Increasing the weight share of additives to 10% increased the modulus by 18%, 55% and 17%, respectively.

### 3.3. Characteristics of Composites Behavior under Compressive Force

**Figure 14:**
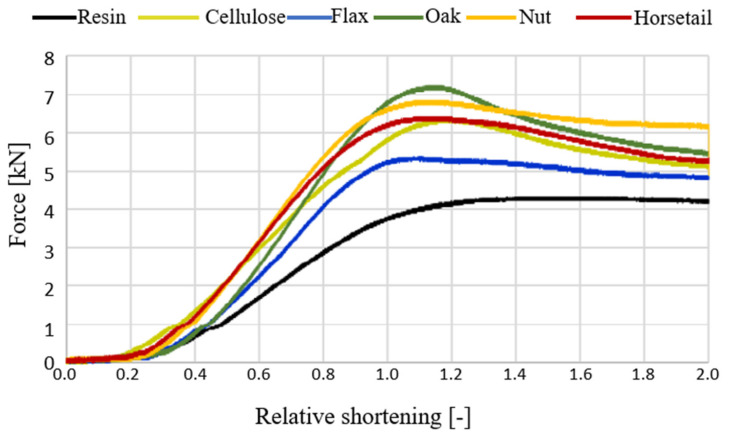
Compression force dependence on relative shortening in the compression test for samples with 2.5% addition.

Figure 14 shows curves of the force vs. relative shortening obtained during the compressive strength test of the produced biocomposites (average values of three samples). Table 2 summarizes the values of the force recorded in specific values of biocomposites’ deformation.

**Figure 15:**
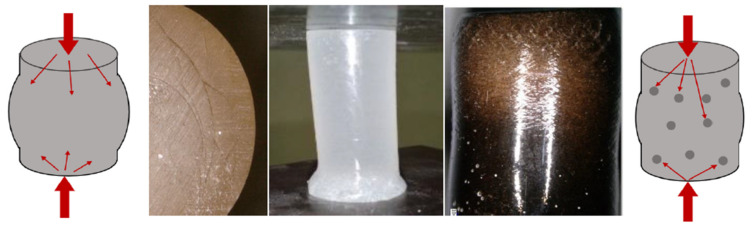
Samples subjected to a compression test.

Figure 14 shows the results of the test, in which the samples with 2.5% modifier content were compressed. It can be concluded that the introduction of even such a small amount of plant additives into the epoxy matrix has a positive effect on the mechanical properties considered in terms of compressive strength. The unmodified resin resists a maximum force of approx. 4 kN, with a deformation of approx. 12%. The introduction of various types of modifiers, which, in the matrix, play the role of a phase that takes over some of the stresses and inhibits the movement of polymer chains, makes the material less deformable. Composite materials effectively resist compressive force up to the deformation of approx. 10%, which translates into force, and increases from 36% to 77% for different additives regarding pure resin. It is worth noting that even for the flax specimens that differed in the strength increase from the others, we observed an improvement in the maximum force of 36%. Among the additives used, oak flour turned out to be the most advantageous for the reinforcement phase. What emerges from the results concerning cellulose content in natural additives reported here is that the influence of modifiers on the strength properties is determined most likely by chemical and material microstructures. In terms of cellulose, the results depend on its content, degree of crystallinity and the angle of the microfiber. In addition, other compounds, such as lignin, or silica for the horsetail cases, are responsible for the strength properties of plant structure. Unfortunately, the results gained do not answer the question about the importance of cellulose’s influence on the additives used on their properties. If we now turn to the failure mode of the specimens, they were not disintegrated and destroyed during the tests. Additionally, few force perpendicular cracks were discovered on sample surfaces. Moreover, bulges of the specimen ends and the color haze due to the accumulation of matrix microcracks (see Figure 15) were present.

Comparing the additives used in terms of the cellulose content, in which the greatest amount is in wood flour, rather than in horsetail (based on the literature “from oak wood < walnut wood”). A hypothesis can be drawn that the increase in the cellulose content in the natural additive makes such composites absorb the impact energy more efficiently.

### 3.4. Strength under Dynamic Loads

**Figure 16:**
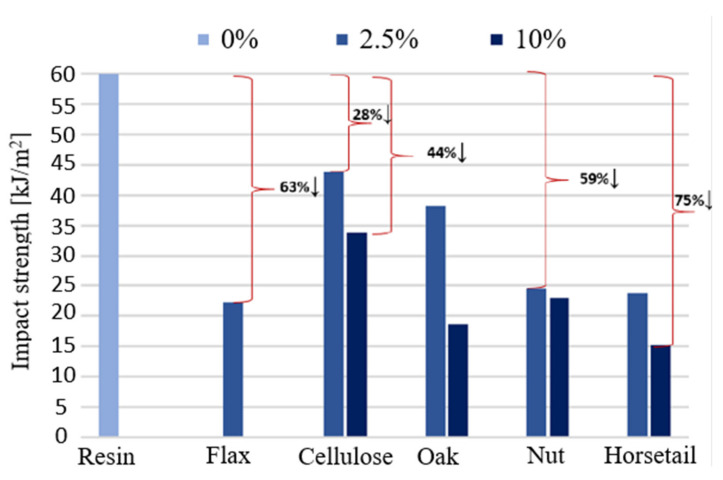
Material impact strength determined by the Charpy method.

Figure 16 shows the results obtained during the impact strength test of the produced biocomposites. The results show that the introduction of even a small amount of the additive, in the form of powder, significantly deteriorates the impact resistance. In the case of using 2.5% of the additive, the smallest changes were recorded for the sample with cellulose. The impact strength decreased by 28%. The biggest changes were observed for the sample with flax fibers and horsetail, a decrease of 63% and 61%, respectively. The more modifiers were introduced, the greater the changes were. Increasing the proportion of the additive to 10% reduced the impact toughness even by 75% (horsetail). This behavior of the material results from the fact that the applied modifying phase (particles) does not act as a reinforcement, which dissipates and absorbs impact energy. The stresses caused by the impact, once they exceed the strength of the composite bond, cause the material to fracture.

### 3.5. Results of Mechanical Tests for Samples with Modified Additives

**Figure 17:**
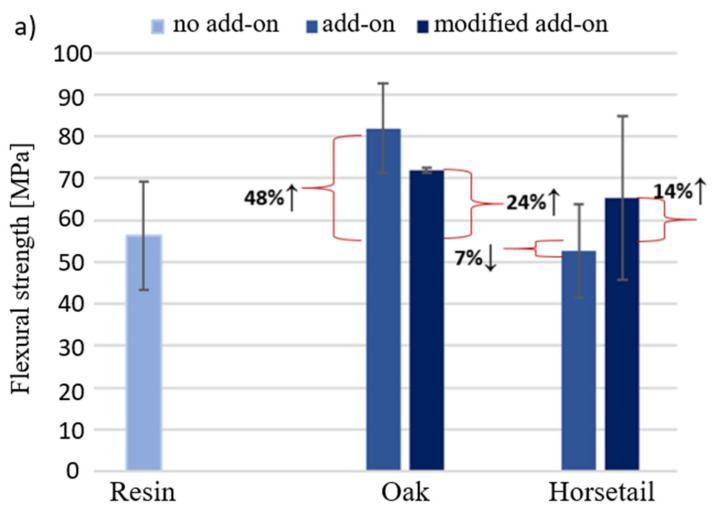

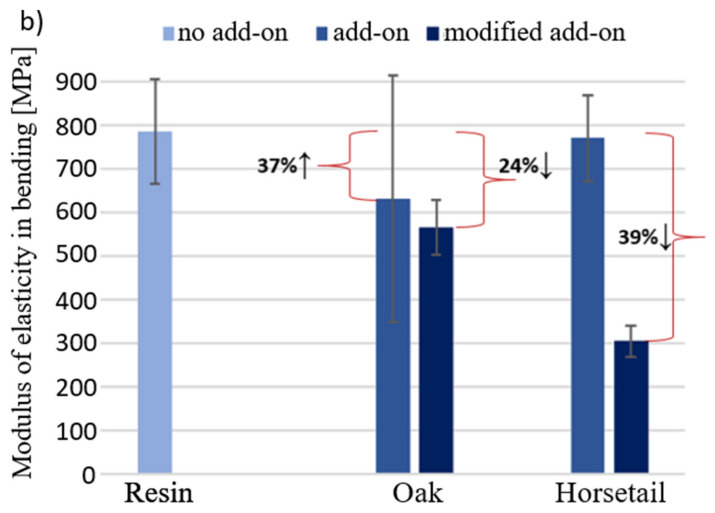
Bending strength (**a**) and modulus of elasticity (**b**) for samples with modified additive.

**Figure 18:**
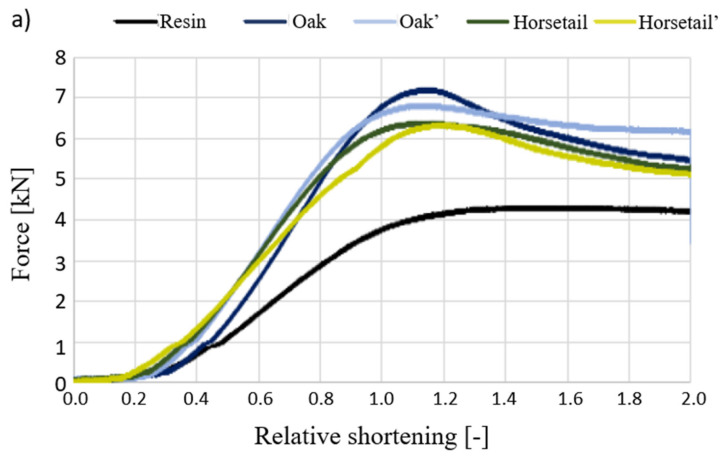

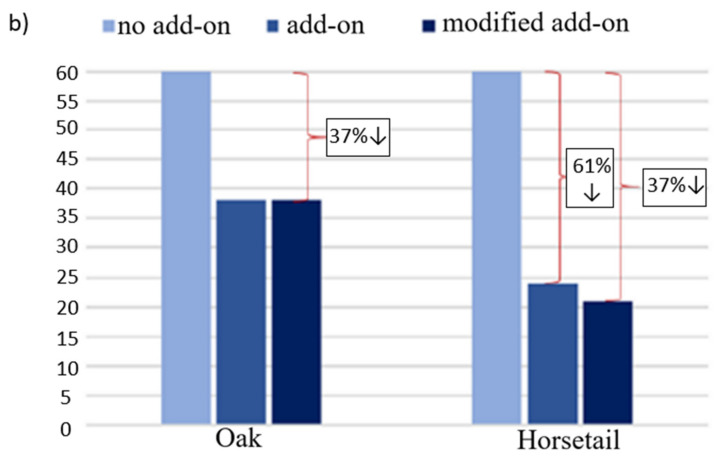
Compression force dependence on relative shortening for samples with modified additives (**a**) and impact strength of materials with modified additives (**b**).

Figure 17 shows the results of the bending strength and modulus of elasticity determined during the bending test of the produced biocomposites containing the modified natural additive. Figure 18 shows the results of the curves obtained during the compressive strength test (a) and the results of the impact strength test (b) of the produced biocomposites containing the modified natural additive.

Turning now to the experimental evidence on the mechanical test results with additives subjected to modifications which depended on lignin removal, in the case of wood flour and carbonization of horsetail, no evidence was found that such treatments significantly affect the properties of the composites (Figure 17 and Figure 18).

Theoretically, the removal of lignin could have a positive effect on the improvement in the mechanical connection of the components by filling the spaces where lignin was previously present with resin. In practice, the results indicate no change or deterioration in properties. The bending strength and modulus of elasticity of the materials with the modified flour, compared to those with the unmodified one, decreased by 12% and 10%, respectively, while the impact strength remained at the same level. However, it should be borne in mind that a small amount of filler was introduced into the composite, and its sedimentation to the bottom of the mold did not allow it to be evenly distributed in the sample, which may affect the reliability of the measurements obtained.

The strength of composites with carbonized horsetail, compared to those with dried horsetail, increased by 24%, its stiffness decreased by 60% and impact strength decreased by 13%.

In the case of compressive strength, no significant changes were recorded for either additive or the other. This proves that what additive and fraction have been used is more important than what treatments it has been subjected to.

### 3.6. Thermal Analysis-Thermal Stability and Mechanism of Thermal Degradation

The analysis of mass changes under the influence of temperature changes was applied to compositions prepared on the basis of epoxy resin with 2.5% and 10% addition of microcrystalline cellulose, flax, wood flour and horsetail. Based on the TG and DTG curves, the thermal stability of the resin used and the influence of the introduced fillers were determined. The temperature at which a 5% weight loss was registered was used to measure thermal stability.

**Figure 19:**
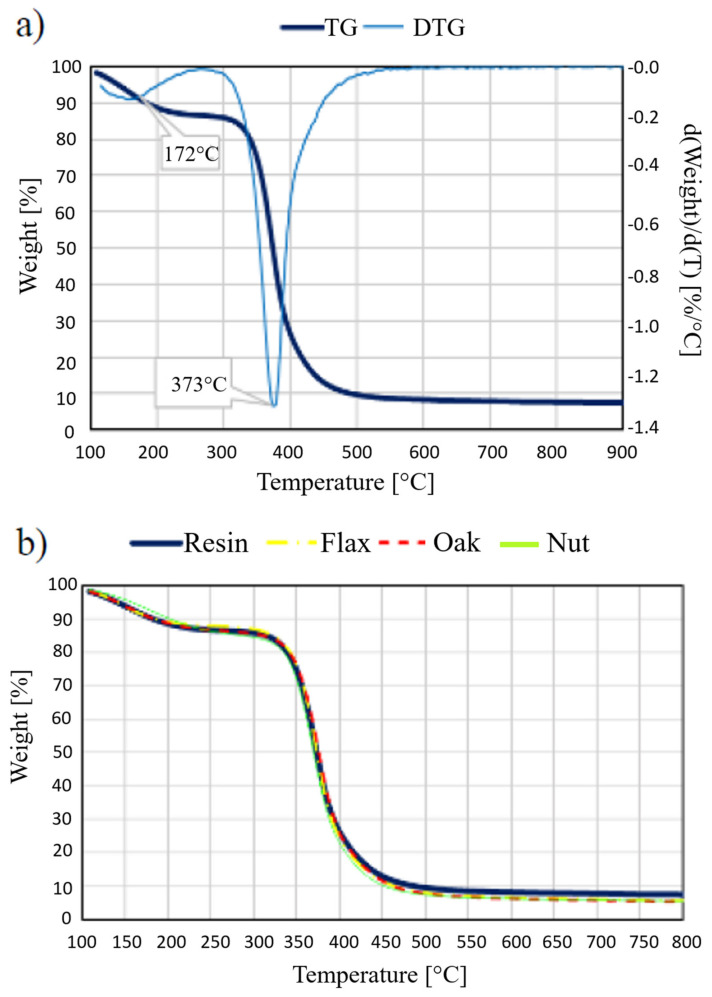
TG and DTG curves for pure resin (**a**), TG curve for resin and composite samples (**b**).

Figure 19a shows the analysis results for pure resin, for which the DTG curve was plotted. On its basis, it can be clearly stated that thermal degradation takes place in two stages. The temperatures with the fastest weight loss are 172 °C for the first stage and 373 °C for the second stage, respectively. The first stage, which runs in the temperature range from about 100 °C to about 230 °C, corresponds to water removal. The second stage, starting at a temperature of about 345 °C and lasting up to 500 °C, corresponds to breaking the bonds of the cross-linked polymer.

Figure 19b compares the course of TG curves of pure resin and composite samples with 2.5% addition of fillers. As can be seen, the incorporation of natural additives into the chemosetting polymer, which is mainly made of cellulose, hemicellulose and lignin, does not significantly affect the thermal stability of the materials obtained from this combination. On the other hand, slightly greater changes in mass for samples with additives can be explained by the fact that natural components undergo thermal degradation (associated with a large loss of mass) at a lower temperature than the duroplastic matrix. These temperatures, for hemicellulose, are from 220 °C and, for cellulose and lignin, from 320 °C.

Exposing the material to the temperature at which the decomposition of the components begins will, in the first place, result in a deterioration of the mechanical properties of the material, due to the formation of microcracks in the matrix around the natural particles. Such a phenomenon will be caused by the fact that plant components will be the first to decompose, the gaseous products of which (CO_2_, CO, H_2_O), having no outlet, will burst the surrounding matrix.

**Figure 20:**
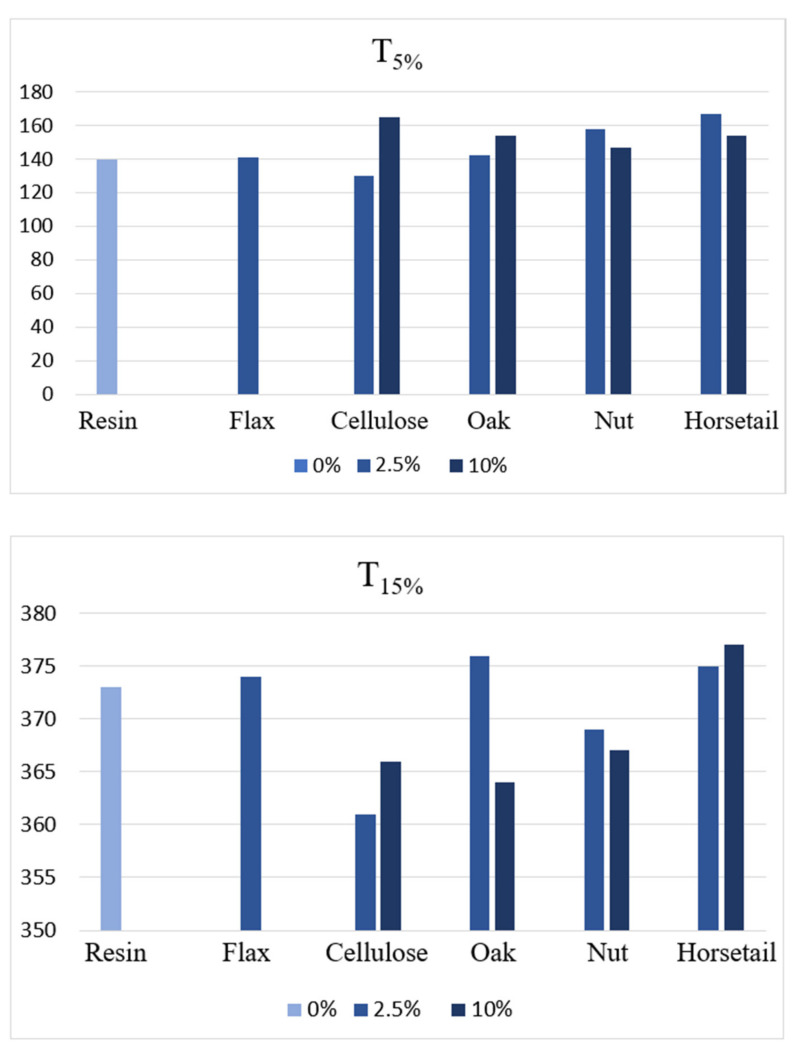
Temperatures in which 5% and 15% weight loss of the sample were registered.

Figure 20 compares the temperatures with 5% and 50% weight loss recorded. For individual samples, greater differences can be noted by analyzing changes for a 5% weight loss. They are visible both in terms of the introduction of a natural additive and its amount. Since such changes occur during the first stage of thermal degradation, they can be explained by the evaporation of water, the content of which, in the sample, may depend on the processes of preparing the components and obtaining materials. In the case of 50% weight loss, which is registered at a temperature of approx. 360 °C, the differences for individual samples are small. This is the temperature at which the polymer matrix and natural components degrade.

On the basis of the obtained results, it is difficult to determine the dependence of the influence of individual natural additives and their amount on the thermal stability of the material, which was defined as a 5% weight loss. For pure resin, it is 140 °C, and the handling of plant components contributed to a slight improvement in temperatures, from 141 °C to 167 °C. Theoretically, in order to increase thermal stability, additives with a high lignin content should be used due to the highest decomposition temperature among the main building components of plant raw materials. The relatively good results for the horsetail samples may be due to the relatively high silica content.

### 3.7. Determination of the Density and Porosity of the Material

The average density of the resin matrix samples is 1.11 ± 0.01 g/cm^3^, which is in line with the parameters provided by the manufacturer. As can be seen, the introduction of natural fillers in the amount of up to 10% (by weight) in relation to the wet ingredients does not significantly affect the density changes (Table 3). Comparing the values of the geometric (actual) density with the values of apparent density, which takes into account the material’s porosity, there are no significant differences, which proves that there is a small presence of pores in the material. However, this is a result that does not accurately reflect the actual state of the samples obtained, in the volume of which, sometimes, quite a significant accumulation of pores could be observed. Obtaining biocomposites with good mechanical properties, which would not be weakened due to defects in the form of air bubbles, is associated with the appropriate optimization of their production process. Moreover, the obtained material is characterized by the negligible presence of open pores and no visible signs of damage on its surfaces.

### 3.8. IR Spectra Analysis

**Figure 21:**
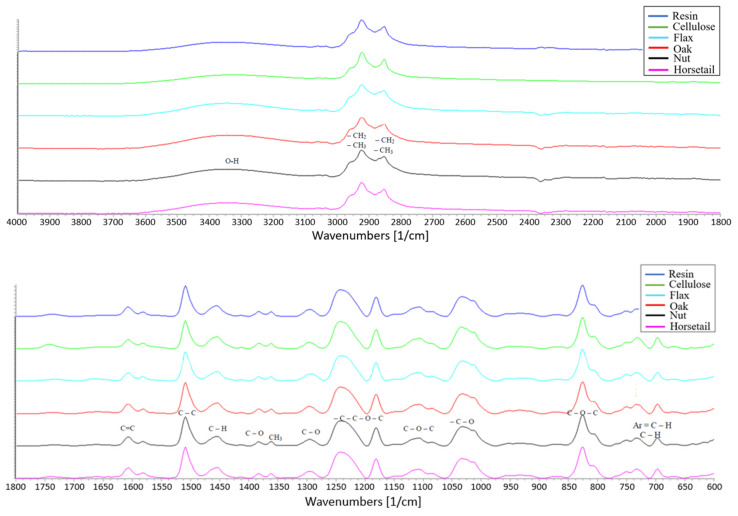
IR spectra of samples with epoxy resin matrix modified with natural additives (upper plot with range of 4000–1800 1/cm, lower plot with range of 1800–600 1/cm.

**Figure 22:**
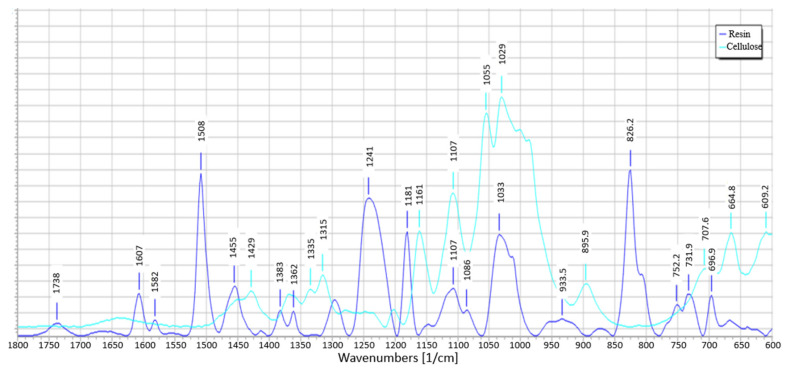
IR spectra for resin and cellulose.

Infrared spectroscopy was performed to analyze the molecular structure of the epoxy resin and to evaluate the effect of the incorporation of high cellulose modifiers (the peaks of all components are already described with specific bonds in Figure 21). Figure 22 shows the specific values of the peak positions which made it possible to identify the bonds. None of the natural additives changes the structure of the materials, which is visible in the spectra, where there is no shift of the bands or additional bands. Such an effect was obtained for samples with a 2.5% modifier share and a 10% share.

## 4. Conclusions

The composites obtained in this study are another example of the possibility of using commonly available and cheap natural resources, such as wood flour and powdered horsetail. Cellulose, which is one of the primary building materials of plant raw materials, is characterized by various forms depending on its source of origin. The properties of the plant walls made of cellulose are determined by the content of the crystalline and amorphous forms and by the angle at which the microfibrils are arranged in relation to the fiber axis. The plant components, whose cellulose contains more of the crystalline phase and the angle of the fibers’ arrangement, is characterized by better features. Based on the results, it was stated:

- Among the natural additives selected to modify the polymer (duroplastic) matrix-microcrystalline cellulose, flax fibers, oak and American walnut wood flour and horsetail—the most favorable characteristics of the biocomposite in terms of bending and compressive strength—were obtained using unmodified oak wood flour. However, it lowered impact resistance of the composite.
- The greatest impact on thermal stability was noted for composites with horsetail, which may be the result of the presence of silica.
- Chemical modification of wood flour did not have a positive effect on the improvement of properties, although it was expected. The test results of the modified additive are not better than that of the unmodified additive. Similar conclusions were drawn from the pyrolytic modification. It allowed for a better crumbling of horsetail and its even distribution in the composite, but the results for the composite are comparable with the unmodified additive.
- Modification of the matrix with components with high cellulose content resulted in improved bending strength. The results of the research indicate that greater improvement was obtained with a lower proportion of plant additives. The introduction of plant additives to the matrix has a positive effect on the improvement of compressive strength. Even a small amount of additive (2.5% by weight of resin + hardener) significantly increased the maximum force that could be resisted by the compressed material (even up to 70% in relation to pure resin). The reinforcement is due to the fact that the particles in the matrix limit the movement of the polymer chains and, thus, the deformation of the matrix.
- The impact strength of composites reinforced with plant particles decreases with the increase in the proportion of the modifying phase. The conducted research shows that the decrease is smaller the more the modifier contains more cellulose, the smaller its fraction and the more even its distribution in the matrix. The exceptions are flax fibers, which contain the most cellulose among plant additives. This is due to the fact that they were introduced in the form of short fibers, in which the homogenization of the components resulted in the accumulation of a significant amount of air, and the production method used turned out to be insufficiently effective for its removal.
- The advantage of introducing natural additives is that it will reduce the formation of toxic substances that are released as a result of the decomposition of the epoxy resin.
- The tests also show that such a combination of components can positively affect the mechanical properties, improving the bending and compressive strength, but lowering the impact toughness.
- The dependence of the improvement/deterioration of properties is a component of several factors, ranging from the modifying additive fraction, its uniformity in the matrix and adhesion between the components, and ending with the type of wood/plant, its chemical composition and microstructure. The next step in the development of duroplastic biocomposites with lignocellulosic additives, especially with a view to assessing the influence of cellulose on properties, could be the introduction of cellulose nanoforms as modifiers. As numerous studies show, even a small addition of nanofibers or cellulose nanocrystals can significantly affect the characteristics of the material.
- The most significant impact on thermal stability was noted for composites with horsetail, which may be the result of the presence of silica.

## Figures and Tables

**Table 1:** Summary of the obtained composites.

Addition/Weight Fraction	Cellulose	Flax	Oak	Oak *	Nut	Horsetail	Horsetail *
2.50%	+	+	+	+	+	+	+
10%	+	−	+	−	+	+	−

* modified material.

**Table 2:** Compressive strength test results.

Relative Shortening [-]	Force F (kN) (Force Corresponding to a Specific Relative Shortening)					
	Resin	Cellulose	Flax	Oak	Nut	Horsetail
0.04	0.7	1.3	0.8	0.8	1	1.3
0.06	1.7	3	2.3	2.6	3.2	3.1
0.08	2.9	4.6	4.1	4.9	5.4	5.1
0.1	3.8	5.8	5.1	6.7	6.6	6.2
Change (%) to the resin at maximum force	-	53↑	36↑	77↑	73↑	63↑

**Table 3:** Results of the measured densities.

Additive Content	Composite	Geometric Density (g/cm^3^)	Apparent Density (g/cm^3^)	Open Porosity (%)
0%	Resin	1.1	1.12	0.08
5%	Flax	1.05	1.08	0.06
	Cellulose	1.12	1.13	0.03
	Oak	1.09	1.13	0.06
	Nut	1.12	1.13	0.06
	Horsetail	1.13	1.13	0.00
10%	Cellulose	1.11	1.15	0.06
	Oak	1.12	1.13	0.03
	Nut	1.11	1.16	0.06
	Horsetail	1.1	1.14	0.06
